# Whole-genome sequencing reveals evidence for inter-species transmission of the yaws bacterium among nonhuman primates in Tanzania

**DOI:** 10.1371/journal.pntd.0012887

**Published:** 2025-02-26

**Authors:** Klára Janečková, Christian Roos, Petr Andrla, Pavla Fedrová, Nikola Tom, Simone Lueert, Julius D. Keyyu, Idrissa S. Chuma, David Šmajs, Sascha Knauf

**Affiliations:** 1 Department of Biology, Faculty of Medicine, Masaryk University, Brno, Czech Republic; 2 Gene Bank of Primates and Primate Genetics Laboratory, Deutsches Primatenzentrum GmbH, Leibniz Institute for Primate Research, Göttingen, Germany; 3 Institute of International Animal Health/One Health, Friedrich-Loeffler-Institut, Federal Research Institute for Animal Health, Greifswald - Insel Riems, Germany; 4 Tanzania Wildlife Research Institute (TAWIRI), Arusha, Tanzania; 5 Tanzania National Parks (Serengeti), Arusha, Tanzania; 6 Professorship for One Health/International Animal Health, Faculty of Veterinary Medicine, Justus Liebig University, Giessen, Germany; Mahidol Univ, Fac Trop Med, THAILAND

## Abstract

**Background:**

*Treponema pallidum* subspecies *pertenue* (*TPE*) is the causative agent of human and nonhuman primate (NHP) yaws infection. The discovery of yaws bacterium in wild populations of NHPs opened the question of transmission mechanisms within NHPs, and this work aims to take a closer look at the transmission of the disease.

**Methodology/Principal Findings:**

Our study determined eleven whole *TPE* genomes from NHP isolates collected from three national parks in Tanzania: Lake Manyara National Park (NP), Serengeti NP, and Ruaha NP. The bacteria were isolated from four species of NHPs: *Chlorocebus pygerythrus* (vervet monkey), *Cercopithecus mitis* (blue monkey), *Papio anubis* (olive baboon), and *Papio cynocephalus* (yellow baboon). Combined with previously generated genomes of *TPE* originating from NHPs in Tanzania (n = 11), 22 whole-genome TPE sequences have now been analyzed. Out of 231 possible combinations of genome-to-genome comparisons, five revealed an unexpectedly high degree of genetic similarity in samples collected from different NHP species, consistent with inter-species transmission of *TPE* among NHPs. We estimated a substitution rate of *TPE* of NHP origin, ranging between 1.77 × 10^-7^ and 3.43 × 10^-7^ per genomic site per year.

**Conclusions/Significance:**

The model estimations predicted that the inter-species transmission happened recently, within decades, roughly in an order of magnitude shorter time compared to time needed for the natural diversification of all tested *TPE* of Tanzanian NHP origin. Moreover, the geographical separation of the sampling sites (NPs) does not preclude *TPE* transmission between and within NHP species.

## Author summary

*Treponema pallidum* subsp. *pertenue* (*TPE*) is the causative agent of neglected tropical disease yaws. This bacterium infects humans in endemic regions in Africa, southern Asia and the Pacific region, and NHPs in Sub-Saharan Africa. The infection manifests as papillomas and/or ulcers, and can progress into deformation of bone and cartilage if untreated. Yaws is targeted by the currently ongoing second yaws eradication campaign, known as the Morges strategy, led by World Health Organization (WHO). In this work, we studied the transmission of the yaws bacterium in wild populations of NHPs, as they could serve as a possible source of reinfection of humans in the future, post-yaws eradication in humans. We have determined eleven whole *TPE* genomes from NHPs from different national parks in Tanzania. Together with previously determined genomes, we have analyzed 22 sequences, and we found a high degree of genetic similarity in samples isolated from different NHP species. This similarity is consistent with inter-species transmission of *TPE* among NHPs.

## Introduction

*Treponema pallidum* subspecies *pertenue* (*TPE*) is the causative agent of yaws in human and nonhuman primates (NHPs) [1–3]. Human yaws is known to be endemic in countries spanning Africa, southern Asia, and the Pacific region [4]. Overall, there are 16 countries currently considered as being endemic for yaws (WHO, https://www.who.int/data/gho/data/themes/topics/topic-details/GHO/yaws-(endemic-treponematoses)). In addition, NHP infection – based on molecular diagnosis and/or visible sightings [2,3,5–9] – has also been documented in several sub-Saharan Africa countries. All countries with infected NHPs were previously known to be endemic for human yaws. While human yaws mainly affects children between the age of 6 to 10, with the main transmission route via close skin contact with an infected person, infection in NHPs in East Africa is generally associated with severe genital lesions indicative of a sexually transmitted disease [10]. However, lesions on the face and extremities also occur, especially in other regions of sub-Saharan Africa, highlighting the potential for the TPE bacterium to also cause both syphilis and yaws-like lesions in its primate host (reviewed in [11]). Until now, *TPE* infections have been diagnosed based on direct detection of the spirochete using darkfield microscopy in *Papio papio* [12] or nucleic acid amplification tests (NAAT) in several species, including *Papio anubis* (olive baboon), *Papio cynocephalus* (yellow baboon), *Chlorocebus pygerythrus* (vervet monkey), *Chlorocebus aethiops* (grivet monkey), *Cercopithecus mitis* (blue monkey), *Gorilla gorilla gorilla* (western lowland gorilla) [3], *Cercocebus atys* (sooty mangabey) [7], and *Pan troglotydes verus* (western chimpanzee) [8]. A previous molecular typing study from Tanzania – the country with the highest number of NHPs sampled – suggested possible inter-species transmission of *TPE* among NHPs based on strain identity [3]. This was further supported by the geographical clustering of samples and the lack of clustering according to species. However, the analysis was limited, with only two gene loci investigated.

In this work, we present eleven genomes of *TPE* isolates that originated from four species of NHPs in four national parks (NPs) in Tanzania. Combined with the 11 previously determined *TPE* genomes derived from Tanzanian NHPs, 22 *TPE* isolates (with determined whole genome sequences) were available for in-depth analysis. Out of 231 possible combinations of genome-to-genome comparisons, five revealed an unexpectedly high degree of genetic similarity between different NHP species, supporting the hypothesis of inter-species transmission of *TPE* among NHPs.

## Materials and methods

### Sample collection

No live animals were sampled for this study. All NHP samples were taken from our previous studies [1,13], and all the necessary ethical statements and DNA extraction details can be found there. Samples for whole-genome sequencing were selected based on (1) the availability of a sufficient treponemal DNA as determined by treponemal copy number via qPCR [2], and (2) the integrity of *TPE* DNA revealed by the amplification of long DNA amplicons [9]. *TPE* DNA integrity was determined by the positivity of long-range PCR that amplified a 4,835 bp-long region encoding the *tprC* gene using primers (ES-42F and TPI-11A-R) and previously published PCR conditions [14]. Selected samples were isolated from four NHP species: *Chlorocebus pygerythrus* (vervet monkey, n = 1), *Cercopithecus mitis* (blue monkey, n = 1), *Papio cynocephalus* (yellow baboon, n = 1) and *Papio anubis* (olive baboon, n = 8). Samples came from Lake Manyara NP (n = 7), Serengeti NP (n = 3), and Ruaha NP (n = 1). Further information about the samples can be found in the Results section.

### DNA target enrichment and whole genome sequencing

For whole genome sequencing, samples with sufficient treponemal DNA and large fragments of *TPE* DNA, indicating the integrity of *TPE* DNA, were selected. Sample 09LMM2180815 was *Dpn*I-enriched according to the protocol published previously by Grillová *et al*. [14] and sent for sequencing to Novogene (Novogene Company Limited, Cambridge, United Kingdom) with the Illumina HiSeq 2500 platform (150 bp paired-end library). All other samples were prepared as per the following protocol. Before sequencing, bacterial DNA was enriched using a Looxter Enrichment Kit (Analytik Jena, Jena, Germany) following the manufacturer’s protocol described in a previous study [9].

DNA target enrichment and library preparation followed the protocols described in a previous study [9]. Briefly, the amplified DNA was purified using SPRISelect beads (Beckman Coulter, Indianapolis, USA). We used the NEBNext Ultra II FS DNA Library Prep Kit for Illumina (New England Biolabs (NEB), Ipswich, USA) with a targeted fragment size of 300–700 bps during the enzymatic digest. Following the ligation of adapters, fragments were size-selected using AMPure XP beads according to the NEB protocol. The library was finalized through single index PCR enrichment of 15 µl adaptor-ligated DNA fragments using a Universal PCR primer (i5 Primer) in combination with the respective Index Primer (i7 Primer). The cycling conditions followed recommendations provided by NEB. Next, PCR reactions were cleaned using AMPure XP beads following standard protocols. Afterwards, quality was checked using a Bioanalyzer 2000 (Agilent, Palo Alto, USA) and quantified using a NEBNext Library Quant Kit for Illumina.

In the next step, *TPE* DNA was enriched using custom-made RNA baits (Arbor Biosciences, Ann Arbor, USA) using the procedure outlined in the myBaits Hybridization Capture for Targeted NGS Manual 4.0 (https://arborbiosci.com/mybaits-manuals/). The bait design is described elsewhere [2]. Following library denaturation and adapter-blocking, target library molecules were allowed to hybridize with their complementary bait. Details of the hybridization process were described previously [9]. In brief, bait-target hybrids were bound to streptavidin-coated magnetic beads that allowed us to separate the desired target DNA molecules from non-target DNA. After magnetic removal of the beads, the supernatant was purified using the SPRIselect clean-up method with NEBNext Sample Purification Beads (x 0.7; New England Biolabs (NEB), Ipswich, USA) according to the NEB protocol. The SPRIselect clean-up product was diluted 5-fold in 0.1X TE buffer and quantified on a Qubit 3.0 (ThermoFisher Scientific, Waltham, USA). In addition, 1 µl of the undiluted SPRI clean-up product was quality-checked on the Bioanalyzer.

Following NEB’s recommended protocol, library quantification was performed using a qPCR NEBNext Quant Kit for Illumina (NEB, Ipswich, USA). We reamplified the library from our first hybridization capture run to increase the yield of target DNA; we used KAPA HiFi Hot Start polymerase in combination with Illumina adapter-specific primers (sense 5’-AAT GAT ACG GCG ACC ACC GA-3’ and antisense 5’-CAA GCA GAA GAC GGC ATA CGA-3’). Cycling conditions were 2 min at 98 °C followed by five cycles of 20 sec at 98 °C, 30 sec at 60 °C, and 45 sec at 72 °C, with the last step being 5 min at 72 °C as the final phase.

The library products were quantified using Qubit 3.0 and cleaned using the SPRIselect method described above; a second round of hybridization capture was performed using the abovementioned method. The enriched 10 nM DNA libraries were pooled, and samples excluding sample 09LMM2180815 were sent for sequencing to the Transcriptome and Genome Analysis Laboratory at the University Medical School Göttingen, Germany. Our workflow included an initial 150 paired-end MiSeq run with the Nano Kit to test for the quality of the enriched libraries and their sequencing performance, prior to in-depth sequencing on Illumina’s HiSeq2500 platform. As a result, majority of samples (except sample 09LMM2180815, 13LMF5300415 and 21F8040407) were sequenced on both MiSeq and HiSeq platforms. S1 Table summarizes the sequencing runs and the GenBank SRA accession numbers.

### Bioinformatic analysis

The bioinformatic analysis was performed using a previously described workflow [9,14]. The genome consensus sequence was determined based on at least three aligned reads (≥3x coverage). Chromosomal loci known to evolve under positive selection [15], genes with extensive intrastrain variability (e.g., *tpr*K and other paralogous *tpr* genes [16,17], genes containing tandem repetitions (e.g., TP0433 and TP0470) and both t0012 and t0015 t-RNA genes (i.e., tRNA-Ala and tRNA-Ile [18]) were removed from the sequence alignments to omit sequences that can emerge either due to positive selection or recombination mechanisms [15,19,20]. These removed sites do not confer any phylogenetic information [14,15,20]. Sequences with these sites removed were used in all subsequent analyses.

Genome sequences were aligned in MEGAX (v.10.2.6, [21]) and used for computing pairwise distances with complete deletion of gap sections. The resulting number of differences (SNPs) in pairwise comparisons of the genomes were then used for further analyses. Bubble plot and boxplots were created in R (R Core Team; Vienna, Austria [22]) using packages readxl [23], ggplot2 [24] and cowplot [25].

### Phylogenetic analysis

Phylogenetic trees were constructed in MEGAX (v.10.2.6). Positions of the alignment containing gaps and missing data were removed for further analyses. For the phylogenetic tree reconstruction of the Tanzanian NHP genomes we used the maximum-likelihood (ML) algorithm using the optimal substitution model as determined by MEGAX. Models used were HKY+G [26] and TN93+G+I [27] used with 1000 bootstrap replicates.

NHP *TPE* genomes from Tanzanian isolates were used (finished genomes, n = 9, shown in bold, and draft sequences, n = 13), as well as complete genomes from African, Indonesian, and Polynesian *TPE* strains isolated from humans [28–31]. *Treponema pallidum subsp. pallidum* (*TPA)* strains Nichols and SS14 [32] were used as outgroup. We inferred an evolutionary history by applying the initial trees for the heuristic search. Initial trees were obtained automatically by applying Neighbor-Joining (NJ) and BioNJ algorithms to a matrix of pairwise distances estimated using the HKY+G model and then selecting the topology with superior log-likelihood values. Additional analysis was performed using HKY+G model with using only 18 *TPE* NHP sequences after removing four draft genomes with the lowest coverage.

Phylogenetic tree (S1 Fig) including draft genome sequences of strains from other available *TPE* genomes of NHP from Cote d’Ivoire, Gambia, Senegal, and Tanzania [2,6,7], and also from *TPE* of human origin, i.e., from Papua New Guinea (specifically Lihir Island), Solomon Islands and Liberia [33–35] were constructed using HKY+G model. Genome sequences were produced from available SRA data using the same approach as described above, except for the genomes originating from the study by Mediannikov (2020) [6], where only draft assemblies are publicly available, and therefore, these draft genome sequences were used for phylogenetic analysis. TPA strains Nichols and SS14 [32] were again used as outgroup. Sequences with less than 57.8% genome coverage were not used for phylogenetic tree reconstructions.

Phylogenetic tree (S6 Fig) using *TPE* NHP sequences of two genes (TP0488 and TP0548) was constructed, using TN93+G+I model [27].

### Evolutionary rate estimation

A ML tree was constructed from the sequence alignment of the *TPE* samples. This tree served as the basis for the subsequent root-to-tip regression analysis using TempEst [36]. We have used Minimizing the Mean of the Squares of the Residuals (RMS) and Maximizing R-Squared approach.

For Bayesian analysis of molecular sequences, BEAST v2.7.4 [37] was used to analyze all 22 *TPE* NHP sequences from Tanzania (with an additional outgroup of the syphilis-causing *TPA* strain SS14 [32] in models T6 and T15). Second analysis was performed on 17 *TPE* NHP sequences after removing four genomes with the lowest coverage and one determined by TempEst (with *TPA* strain SS14 as an outgroup in models T6 and T15). For the analysis, we removed all positions with gaps and missing sequence data from the alignment. Tip dates were specified based on the years of sample collection. To assess model fit, we employed Nested Sampling in BEAST2. To infer the evolutionary history, we employed the HKY model and the strict clock model in combination with three other different models, including the coalescent constant population demographic model (T5), the birth-death model (T6), and the Yule model (T15). The BEAST analysis was run for 10 million Markov chain Monte Carlo (MCMC) generations with a 10% burn-in. We combined results from three runs with LogCombiner 2.4 [37], resulting in 27 million computed generations. Parameter estimates were obtained by Tracer v1.7.2 [38], and a maximum clade credibility tree was generated with TreeAnnotator v2.7.4 [37]. The resulting tree was visually represented in FigTree v1.4.4 [39]. Two NHP *TPE* whole genome sequences (without putative recombinant sites, positively selected sequences, repetitions, and *tpr* sequences) of most closely genetically related strains were selected for the estimation of substitution rates in NHP *TPE*.

## Results

### Characteristics of eleven nonhuman primate *TPE* whole genomes

The Tanzanian NHP *TPE*-genomes newly generated in this study (n = 11) are shown in Table 1. The final coverage of these genomes ranged from 57.8% to 98.9% (with a sequencing depth of ≥3×), and the average sequencing depth ranged from 4× to 116× (Table 1).

**Table 1:** Metadata of nonhuman primate *Treponema pallidum* subspecies *pertenue* isolates analyzed in this study and the resulting whole-genome sequencing parameters.

*TPE* isolate	Source	Sex	Origin	Year of isolation	Genome coverage (%)	Average sequencing depth
2SNF2130815	*Papio anubis*	F	SNP	2015	98.71	116×
6SNF2081115	*Papio anubis*	F	SNP	2015	98.80	34×
13LMF5300415	*Papio anubis*	F	LMNP	2015	74.70	9×
21LMF2290815	*Papio anubis*	F	LMNP	2015	57.80	4×
21F8040407	*Papio anubis*	F	LMNP	2007	78.10	9×
50F2190407	*Papio anubis*	F	LMNP	2007	87.30	13×
63M8270407	*Papio anubis*	M	LMNP	2007	77.10	8×
70M5100507	*Papio anubis*	M	LMNP	2007	98.88	46×
14RUF5130716	*Papio cynocephalus*	F	RNP	2016	98.89	93×
09LMM2180815	*Cercopithecus mitis*	M	LMNP	2015	97.90	30×
41SNM2231115	*Chlorocebus pygerythrus*	M	SNP	2015	93.50	28×

M – male, F – female; SNP - Serengeti National Park, TZ; LMNP - Lake Manyara National Park, TZ; RNP - Ruaha National Park, TZ.

**Fig 1:**
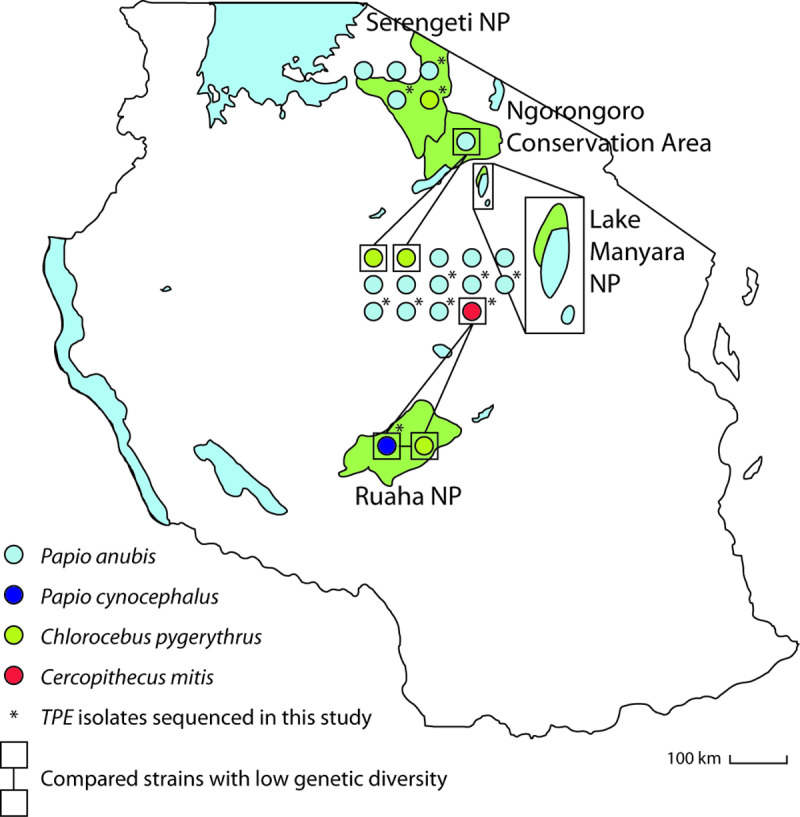
A schematic map of Tanzania with relevant national parks and conservation areas linked to this study. National parks and the Ngorongoro Conservation Area are shown in green. Whole genome sequences of NHP *TPE* isolates from Tanzania are shown as circles. Circle fill color corresponds to NHP species. Inter-species comparisons with low levels of genetic diversity (as determined later) are shown in boxes. Made with Natural Earth (base layer available at https://www.naturalearthdata.com/features/).

A geographical representation of the *TPE*-genome origins (n = 11) and the previously published *TPE* genomes (n = 11) [2,9] are shown in Fig 1. Most of the samples originated from Lake Manyara NP (n = 14), followed by Serengeti NP (n = 5), Ruaha NP (n = 2) and Ngorongoro Conservation Area (n = 1).

### A pairwise comparison of whole genome *TPE* sequences

**Fig 2:**
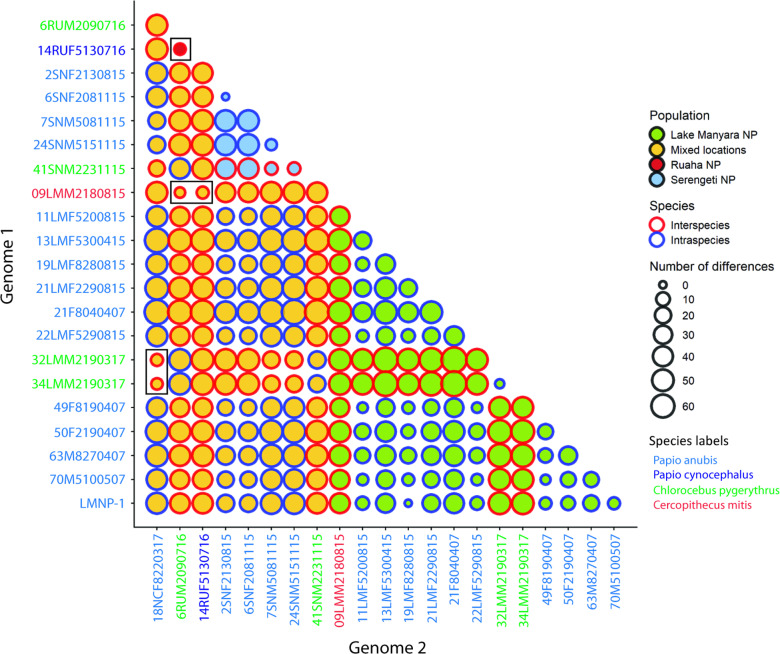
A schematic representation of pairwise whole genome comparisons in all possible pairwise combinations (n **=** **231) based on multiple sequence alignments of Tanzanian NHP *TPE* genome sequences.** Complete gap deletion algorithm was used. There was a total of 386,092 positions in the final dataset. Exact numbers of detected nucleotide differences can be found in S3 Table. The size of the circles denotes the number of detected nucleotide differences between pairs of genomes, and the fill color corresponds to the population in a particular sampling area. The red outer circle color denotes the comparison of *TPE* isolates obtained from different species, while the blue outer circle denotes NHP intraspecies comparisons. Color of the axes labels corresponds to NHP species. Small circles with red rings correspond to interspecies comparisons with nearly identical genome sequences (boxed).

A pairwise comparison of whole NHP *TPE* genome sequences of Tanzanian strains is shown in Fig 2. All possible pairwise combinations (n = 231; 22×21/2) were tested for nucleotide differences. The median number of detected nucleotide differences (n = 41) was relatively high; however, in some cases, the number of differences dropped to zero (Fig 2).

### Values of pairwise combination differences discovered through whole genome sequence comparisons

**Fig 3:**
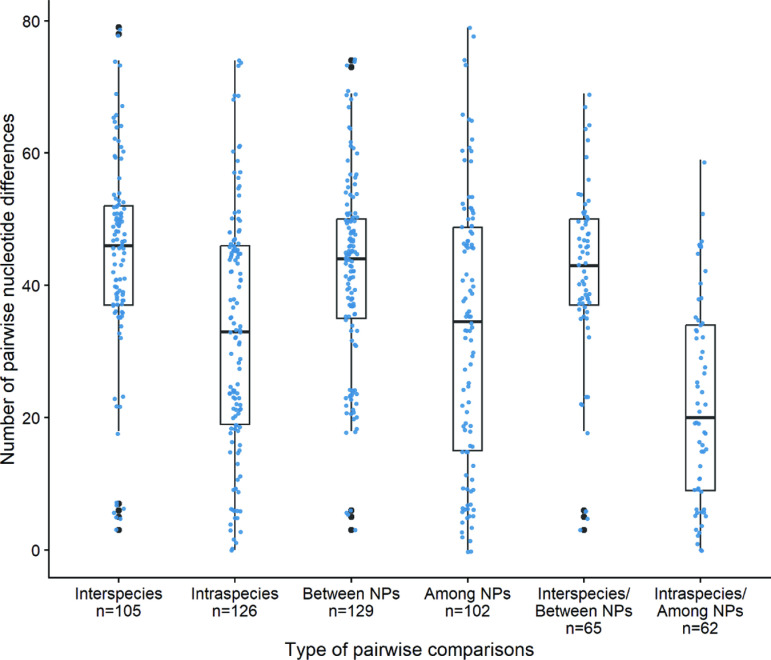
Box plot analysis of values of pairwise nucleotide distances between *TPE* genomes. While the median of all distances was 41 nucleotides, inter-species and inter-national park distances were higher compared to intra-species and within national park differences, respectively. The lower and upper hinges (edges) correspond to the first and third quartiles (i.e., the 25th and 75th percentiles). Data points beyond the whiskers are considered outliers and plotted individually.

**Fig 4:**
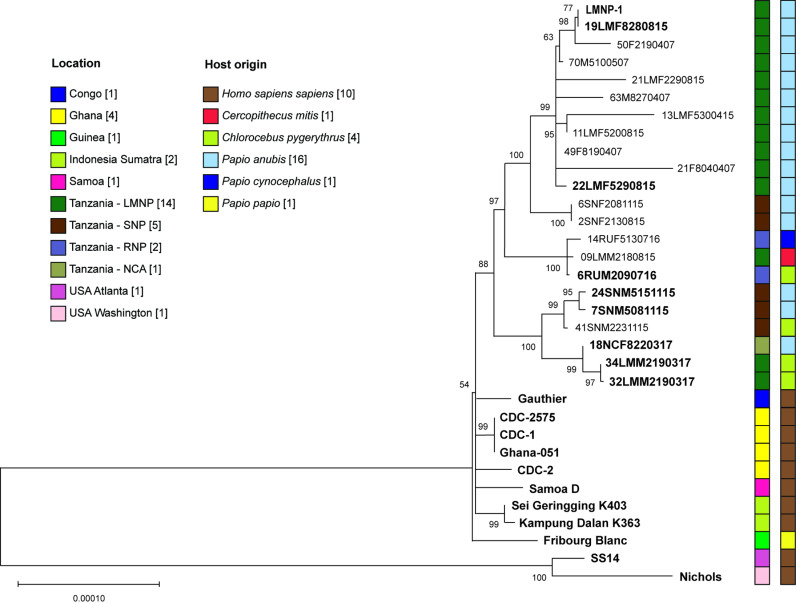
Phylogenetic analysis of Tanzanian NHP *TPE* samples. NHP *TPE* genomes from Tanzanian isolates used were either finished genomes (n = 9, shown in bold) or partially determined (draft sequences, **n** = 13), as well as completely sequenced genomes from African, Indonesian, and Polynesian *TPE* strains isolated from humans [28–31]. TPA strains Nichols and SS14 [32] were used as an outgroup. The evolutionary history was inferred using the Maximum likelihood and HKY+G model [26]. All positions containing gaps and missing data were eliminated (complete deletion option). There were 1,981 variable sites in the original dataset. There were 385,875 positions in the final dataset (35% of the whole genome (1,093 kb)). Bootstrap support (1000 replicates) is shown next to the branches. The scale corresponds to the number of substitutions per nucleotide. The geographical and species origin of isolates is shown next to the tree. Finished genomes are shown in bold.

All of the values of pairwise differences determined with complete deletion option (386,092 nt positions in the final dataset) are shown in S3 Table. The values that were obtained using pairwise deletion option can be seen in S4 Table. The length of the genomes used for the pairwise comparisons can be found in S5 Table. The median value of pairwise differences was 41 nucleotides between pairs of genomes with a range from 0–79 (Fig 3). Inter-species combinations showed a higher median value (med(x) = 46) compared to the intra-species median value (med(x) = 33). Similarly, a difference was found between pairwise differences derived from the same sampling site (med(x) = 34.5) compared to pairwise differences coming from different sites (different NPs; med(x) = 44). When both comparisons were combined, the differences were even higher (Fig 4), i.e., the median number of nucleotide differences for inter-species comparisons in different locations (med(x) = 43) was higher compared to intra-species comparisons from the same site (med(x) = 20).

### A pairwise comparison of *TPE* genomes obtained from different NHP species

The pairwise comparison of all analyzed *TPE* genomes isolated from different NHP species allowed us to identify genomes with the lowest number of nucleotide differences (Table 2). Among five such genome comparisons, the number of differences ranged between 3–6 nucleotides when comparisons were based on a complete gap deletion algorithm using multiple alignments where only nucleotide positions present in all genomes were compared (386,092 nt positions in the final dataset). In the pairwise comparisons based on the *de novo* alignment of every two sequences, the number of detected nucleotide differences was higher and ranged between 10–17 (corresponding to a genome sequence identity of 99.9991%–99.9985%), and the number of amino acid replacements in the corresponding proteome ranged between five and ten. The genes showing differences between highly related *TPE* samples and the corresponding amino acid changes are shown in S2 Table.

**Table 2:** Comparison of *TPE* genomes isolated from different NHP species with the lowest pairwise differences. The number of detected single nucleotide and amino acid differences in the predicted proteins are shown.

Sample 1	Sample 2	Year of isolation sample1/2	No. of nt differences (complete deletion*)	No. of nt differences (partial deletion**)	Number of aa differences***	Location 1	Location 2	Species 1	Species 2
09LMM2180815	6RUM2090716	2015/2016	3	11	9	LMNP	RNP	*C. mitis*	*C. pygerythrus*
09LMM2180815	14RUF5130716	2015/2016	6	15	10	LMNP	RNP	*C. mitis*	*P. cynocephalus*
6RUM2090716	14RUF5130716	2015/2016	5	17	10	RNP	RNP	*C. pygerythrus*	*P. cynocephalus*
32LMM2190317	18NCF8220317	2017/2017	6	11	6	LMNP	NCA	*C. pygerythrus*	*P. anubis*
34LMM2190317	18NCF8220317	2017/2017	5	10	5	LMNP	NCA	*C. pygerythrus*	*P. anubis*

aa, amino acid; LMNP, Lake Manyara National Park, TZ; RNP, Ruaha National Park, TZ; NCA, Ngorongoro Conservation Area, TZ.

*pairwise comparisons based on complete gap deletion algorithm in multiple alignment.

**pairwise comparisons based on *de novo* alignment of two sequences.

***amino acid differences in the predicted proteins

### Phylogenetic analysis

The whole genome NHP *TPE* sequences newly generated in this study (n = 11), augmented with additional NHP *TPE* genomes from Tanzania (n = 11; [2,9]), were analyzed in the context of other available NHP and human *TPE* genomes. Despite the relatively low bootstrap support, all 22 isolates from Tanzania analyzed to date clustered separately from the twelve NHP *TPE* genome sequences from strain/isolates originating in West African countries, i.e., Senegal, Gambia, Guinea, and Côte d’Ivoire [2,5,6] (S1 Fig). The phylogeny of the Tanzanian NHP *TPE*-genomes together with the Fribourg-Blanc strain that infects NHPs in West Africa, as well as selected complete *TPE* genomes from African, Indonesian, and Polynesian strains of human origin, is shown in Fig 4. We note here that several whole genome sequences were nearly identical (e.g., 32LMM2190317 and 34LMM2190317, both derived from samples from *Chlorocebus pygerythrus,* with one nucleotide difference, corresponding to several years of separate evolution) and some were isolated from different NHP species (e.g., 6RUM2090716 (*Chlorocebus pygerythrus*) and 09LMM2180815 (*Cercopithecus mitis*)). Additional phylogenetic tree (S2 Fig) with four lowest coverage genomes removed has also been calculated.

### Estimated substitution rates revealed by comparison of the most closely related genomes and BEAST analysis of genomic sequences

In this study, we used TempEst to perform root-to-tip regression analysis on a dataset comprising 22 *TPE* samples from Tanzania (S3 and S4 Figs). The dataset spans a 10-year collection period, allowing us to evaluate the consistency of the temporal signal across the samples. We conducted the BEAST analysis twice: first, with all 22 samples included, and second, with 17 samples after exclusion of four samples exhibiting the lowest average sequencing depth and one other outlying sample as determined by TempEst. These two analyses aimed to investigate the impact of low-quality data on the inferred evolutionary rates and the robustness of the temporal signal.

Two *TPE* whole genome sequences, including LMNP-1 (sample isolated in April 2007; [2]) and 19LMF8280815 (sample isolated in August 2015; [9]), both coming from olive baboons at Lake Manyara NP, were selected for the estimation of substitution rates in NHP *TPE*. Both genomes differed in two nucleotides when whole genomes without putative recombinant sites, positively selected sequences, repetitions, and *tpr*K sequences were used, respectively. If both strains evolved from a common ancestor, the corresponding substitution rate would be equal to 2.20 × 10^-7^ per genomic site per year (2 nt differences between genomes; 1,092,306 and 1,092,317 nt genome length for 19LMF8280815 and LMNP-1, respectively; both *TPE* were isolated 8.33 years apart). In addition, BEAST analysis of all 22 NHP *TPE* sequences from Tanzania was used to estimate substitution rates using BEAST models T5, T6, and T15 (with an outgroup of *TPA* SS14 [32] used in models T6 and T15). In this analysis, the resulting length of analysed sequence was 386,063 nt (35% of the whole genome (1,093 kb)). This revealed estimated median substitution rates of 3.43 × 10^-7^ (95% HPD 5.16×10^-10^ – 8.64×10^-7^), 2.22 × 10^-7^ (95% HPD 7.41×10^-9^ – 5.17×10^-7^), and 2.76 × 10^-7^ (95% HPD 7.21×10^-9^ – 6.39×10^-7^), respectively (Table 3 and S5 Fig). Using BEAST models, these substitution rates were used to further estimate the elapsed time from the most recent common ancestor (Table 3). The lowest estimated times from the most recent common ancestor of *TPE* isolated from different species ranged between 51.3 and 99.3 years, while the estimated time from the most recent common ancestor of the most divergent *TPE* from Tanzanian NHPs ranged between 528.2 and 987.5 years.

**Table 3:** BEAST analysis of all 22 NHP *TPE* sequences from Tanzania (with the *TPA* SS14 outgroup in T6 and T15 models). Estimated substitution rates and estimated elapsed time from the most recent common ancestor using BEAST models T5, T6, and T15.

Method for estimation of substitution rate	Estimated substitution rate in nucleotide changes per site and year (95% HPD* interval)	Comparison of 09LMM2180815/ 6RUM2090716 (years to common ancestor)	Comparison of 34LMM2190317/ 18NCF8220317 (years to common ancestor)	Maximal estimated time span among 22 *TPE* NHP (years to common ancestor)
BEAST T5 model (22 *TPE* NHP sequences)	3.43 × 10^-7^ (5.16×10^-10^ – 8.64×10^-7^)	88.7	99.3	987.5
BEAST T6 model (22 *TPE* NHP sequences)	2.22 × 10^-7^ (7.41×10^-9^ – 5.17×10^-7^)	57.6	69.1	620.7
BEAST T15 model (22 *TPE* NHP sequences)	2.76 × 10^-7^ (7.21×10^-9^ – 6.39×10^-7^)	51.8	61.9	528.2

*highest posterior density interval

To further extend our analysis, we performed the same BEAST analysis while removing five sequences of NHP *TPE* with the lowest coverage, resulting in 874,228 nt genome length alignment used for analysis (80% of the whole genome (1,093 kb)). This additional analysis revealed estimated median substitution rates of 1.77 × 10^-7^ (95% HPD 1.27×10^-10^ – 4.00×10^-7^), 1.81 × 10^-7^ (95% HPD 1.03×10^-8^ – 3.77×10^-7^), and 2.29 × 10^-7^ (95% HPD 2.23×10^-8^ – 4.73×10^-7^), respectively (Table 4). Using BEAST models, these substitution rates were used to further estimate the elapsed time from the most recent common ancestor (Table 4). The lowest estimated times from the most recent common ancestor of *TPE* isolated from different species ranged between 31.4 and 121.0 years, while the estimated time from the most recent common ancestor of the most divergent *TPE* from Tanzanian NHPs ranged between 393.5 and 1148.0 years.

**Table 4:** BEAST analysis of 17 NHP *TPE* sequences from Tanzania (with the *TPA* SS14 outgroup in T6 and T15 models). Estimated substitution rates and estimated elapsed time from the most recent common ancestor using BEAST models T5, T6, and T15.

Method for estimation of substitution rate	Estimated substitution rate (nucleotide changes per site and year)	Comparison of 09LMM2180815/ 6RUM2090716 (years to common ancestor)	Comparison of 34LMM2190317/ 18NCF8220317 (years to common ancestor)	Maximal estimated time span among 22 *TPE* NHP (years to common ancestor)
BEAST T5 model (17 *TPE* NHP sequences)	1.77 × 10^-7^ (1.27×10^-10^ – 4.00×10^-7^)	121.0	82.6	1148.0
BEAST T6 model (17 *TPE* NHP sequences)	1.81 × 10^-7^ (1.03×10^-8^ – 3.77×10^-7^)	55.9	38.8	515.2
BEAST T15 model (17 *TPE* NHP sequences)	2.29 × 10^-7^ (2.23×10^-8^ – 4.73×10^-7^)	45.2	31.4	393.5

*highest posterior density interval

## Discussion

Today, Tanzanian NHP *TPE* genomes represent the majority of the available NHP *TPE* sequence data compared to the twelve NHP *TPE* draft genome sequences coming from NHPs sampled in Senegal, Gambia, Guinea, and Côte d’Ivoire [2,5,6]. Our recent work contributes another eleven NHP *TPE* genomes of Tanzanian origin. Combined with the previously determined NHP *TPE* genomes from the same region [2,9] a total of 22 *TPE* genome sequences from four NHP species (sampled at four different locations between 2007 and 2017) are now available. A previous multi-locus sequence and phylogenetic network analysis of *TPE* strains infecting African primates, including the above-stated areas, revealed a geographic clustering of analyzed *TPE* strains [2,3]. Together with the absence of clustering according to host species, it suggests ongoing inter-species transmission at the local level. To further test this hypothesis, *TPE* genome sequences from Tanzanian NHPs were analyzed regarding the presence and degree of genomic diversity. Unlike the previous work [9], where the *TPE* sample set was selected based on genetic diversity revealed from two gene loci (TP0488 and TP0548 [3]) and was therefore of limited importance to address this question, in this work we selected samples based on the availability of samples and their quality (i.e., DNA integrity). Despite the limited number of samples (n = 22), our current sample set represents an unbiased collection for investigating the genetic diversity among the different NHP-derived *TPE* genomes.

As revealed by the phylogenetic analysis, the geographical separation of sampling areas within Tanzania is not entirely unambiguous and, therefore, different geographical locations do not appear to limit transmission of *TPE* within Tanzania (e.g., Ruaha NP vs Lake Manyara NP, ~360 km distance). However, as expected, the genetic relationship within the Tanzanian NHP *TPE* strains is closer than that between Tanzanian NHP-derived genomes and those infecting NHPs in West Africa (S1 Fig). The same effect is equally visible at the local scale. Analyses of genetic diversity based on whole genome alignments within the four sampled national parks in Tanzania, for example, revealed lower genetic diversity within the geographic sampling locations compared to the genetic diversity calculated for *TPE* comparisons between different parks (Fig 3). This suggests that the epidemiological spread of *TPE* is fastest on the local scale, with a lower rate of spread across the sampling sites, and within the African continent.

The nature of the *TPE*-host system can explain our findings. The bacterium, for example, does not survive for long in a non-host environment [31], limiting the spread, e.g., through flies as potential mechanical vectors [40,41]. Although modern, well-characterized experimental data are missing, all current data, i.e., human infections, suggest close skin-to-skin (sexual and non-sexual) contact as the main route of infection. Moreover, NHPs are relatively resident, and migration rarely happens over longer distances [42], explaining the slower spread of the disease across the different regions in Tanzania. In addition to geographic barriers in *TPE* transmission, similar analysis within and between different NHP species revealed a similar difference in genetic diversity, supporting our previous work that the disease in Tanzanian NHPs is mainly sexually transmitted [1]. However, sexual behavior in NHPs is a porous species barrier to *TPE* transmission, as shown, for example, by the inverted intergeneric introgression between the critically endangered kipunjis (*Rungwecebus kipunji*) and yellow baboons (*Papio cynocephalus*) in two disjunct populations in Tanzania [43]. Additionally, different primate species can form mixed-species groups [44–46], and interaction between the individuals of different species, either aggressive (hitting, biting, leg or tail-pulling) or playful, including grooming activities, is a well-documented phenomenon [47–49].

As expected, highly related samples were found when the same species were sampled in one area at the same time (e.g., 32LMM2190317 and 34LMM2190317 differing in just one nucleotide, [9]; 2SNF2130815 and 6SNF2081115 that are genetically identical in the analyzed sequences, this study). Infection with the same (or a highly related) *TPE* strain is, therefore, common in a population at a given time. This aligns with our previous work, where we could show, based on MLST, that the number of strains infecting olive baboons is limited and, at Lake Manyara NP, ranges between six in 2007 and five in 2015 [3]. Surprisingly, genetically highly related samples were also found when different NHP species were sampled in one area simultaneously or in different NPs at different time points. Previous predictions on inter-species transmission between different NHP species were based on analyses of two chromosomal loci (TP0488 and TP0548) [3]; however, these loci were found to be recombinant and positively selected in *TPA* and *Treponema pallidum* subsp. *endemicum* (*TEN)* treponemes [15,19,20,50,51]. The phylogenetic tree based on analysis of these two loci differed from the tree based on whole genome sequences (S6 Fig); however, due to the low bootstrap support of the two-loci-based tree, it is not clear how the difference between the trees is relevant. The convergent evolution of these loci could explain the observed similarities based on these two chromosomal sequences. Since analyses based on whole genome sequences do not allow this explanation, highly related *TPE* isolates infecting different NHP species provide clear evidence for direct inter-species transmission of *TPE* strains.

We have used several methods and models to estimate the substitution rate of NHP *TPE*. These estimates ranged between 1.77 × 10^-7^ and 3.43 × 10^-7^ per genomic site per year, varying slightly more than by a two-fold difference. By analysis of genetically related *TPE* strains isolated at different time points and propagated in laboratory animals, substitution rates of human *TPE* isolates have been estimated to be 1.21 × 10^−7^ per nucleotide site per year or lower [52]; the substitution rate of *TPA* has been estimated to be 0.82 × 10^−7^ per nucleotide site per year or lower [53], using two TPA isolates one of which was propagated in rabbits. However, these values are very close to the rates estimated in this study. Moreover, the estimated substitution rates in other studies based on alignments of draft genomes of *TPA* sequences were equally similar to our estimated rates, i.e., 3.02 × 10^−7^ [54], 6.6 × 10^−7^ [55], and 1 × 10^−7^ [56] per nucleotide site per year. However, substitution rate estimates are likely subject to variation across different *T. pallidum* subspecies and strains that infect humans and, in the case of *TPE*, also NHPs. Nevertheless, our estimations of substitution rates allow us to predict that the inter-species transmission among Tanzanian NHPs happened in recent history, in the order of decades (31.4 – 121.0 years), which is about an order of magnitude faster than the time interval needed for natural diversification of all known *TPE* strains that infect Tanzanian NHPs (393.5 – 1148.0 years) as estimated by our BEAST analysis.

While this study provides further proof for inter-species transmission of *TPE* among NHPs, the potential transmission of the yaws bacterium from NHPs to humans remains hypothetical. Our investigations of humans in areas in Tanzania where NHPs are infected found no indication of yaws infection [57]. Unfortunately, no samples or data are available from the time when Tanzania reported its largest number of human infections, i.e., between 1930 and 1950 [58]. These data would help to trace the origin of human infections. A clear obstacle for current *TPE* research is the scarcity of available samples or data from one geographic area at a given period. Our work shows ongoing inter-species transmission in primates, a mammalian order taxon that also includes humans. This underlines the need to continue disease surveillance in humans that co-exist with infected NHPs even where yaws has been eliminated in humans. Any undetected – though rare – spillover could potentially result in a re-emergence of yaws in humans. More research, using complete genomes, is warranted in sub-Saharan Africa to investigate the relationship between NHPs and humans relative to *TPE* infections.

## Supporting information

S1 Table Overview of samples and sequencing platforms used, including GenBank SRA and Genome accession numbers of the BioProject No. PRJNA1062612.
(XLSX)

S2 Table Comparison of *TPE* genomes isolated from different NHP species.
The number of detected single nucleotide variants (SNVs), amino acid changes, and affected genes are shown.
(XLSX)

S3 Table Pairwise comparison of *TPE* genomes using the complete deletion algorithm.
Numbers of detected single nucleotide variants (SNVs) using complete deletion.
(XLSX)

S4 Table Pairwise comparison of *TPE* genomes using the pairwise deletion algorithm.
Numbers of detected single nucleotide variants (SNVs) using pairwise deletion.
(XLSX)

S5 Table Pairwise comparison of *TPE* genomes using the pairwise deletion algorithm – length of the genome used for each comparison.
(XLSX)

S1 Fig Phylogenetic analysis of all available *TPE* genomes isolated from NHPs or humans.
Draft genomes available from other studies were used [2,6,7,33–35]. Sequences with less than 57.8% genome coverage were not used for phylogenetic tree reconstructions. All positions containing gaps and missing data were deleted. The evolutionary history was inferred using the Maximum Likelihood method and HKY+G model [26]. As an outgroup, human syphilis genomes of strains Nichols and SS14 [32] were used. There were 93 nucleotide sequences and a total of 256,792 positions in the final dataset. Bootstrap support (1000 replicates) is shown next to the branches. The scale corresponds to the number of substitutions per nucleotide. *TPE* genomes from Tanzanian NHP isolates clustered together.
(TIF)

S2 Fig Phylogenetic analysis of 18 Tanzanian NHP *TPE* samples.
NHP *TPE* genomes from Tanzanian isolates. Completely sequenced genomes from African, Indonesian, and Polynesian *TPE* strains isolated from humans are also shown [28–31]. As an outgroup, human syphilis genomes of strains Nichols and SS14 [32] were used. The evolutionary history was inferred using the Maximum likelihood and HKY+G model [26]. All positions containing gaps and missing data were eliminated (complete deletion option). There were 873,701 positions in the final dataset. Bootstrap support (1000 replicates) is shown next to the branches. The scale corresponds to the number of substitutions per nucleotide.
(TIF)

S3 Fig Root-to-Tip Regression with Minimizing RMS (Residual mean squared).
A. Phylogenetic tree. B. Root-to-tip regression graph.
(TIF)

S4 Fig Root-to-Tip Regression with Maximizing R-Squared. A. Phylogenetic tree. B. Root-to-tip regression graph.
(TIF)

S5 Fig Phylogenetic analysis of Tanzanian *TPE* samples isolated from NHPs analyzed using the BEAST T5 model.
The human syphilis SS14 genome [32] was used as an outgroup. The estimated time elapsed from the most common ancestor is shown next to the branches. The length of the scale bar corresponds to 1000 years.
(TIF)

S6 Fig Phylogenetic analysis of Tanzanian *TPE* samples isolated from NHPs based on two genes (TP0488 and TP0548).
All positions containing gaps and missing data were deleted. The evolutionary history was inferred using the Maximum Likelihood method and TN93+G+I model [27]. As an outgroup, the human syphilis strains Nichols and SS14 genome [32] were used. There were 33 nucleotide sequences and a total of 1,714 positions in the final dataset. Bootstrap support (1000 replicates) is shown next to the branches. The scale corresponds to the number of substitutions per nucleotide.
(TIF)
