# Supplementary figures and images for: Whole-genome sequencing reveals evidence for inter-species transmission of the yaws bacterium among nonhuman primates in Tanzania
Source: PLoS Negl Trop Dis. 2025 Feb 26;19(2):e0012887. doi: 10.1371/journal.pntd.0012887 (PMC11864524; doi:10.1371/journal.pntd.0012887)

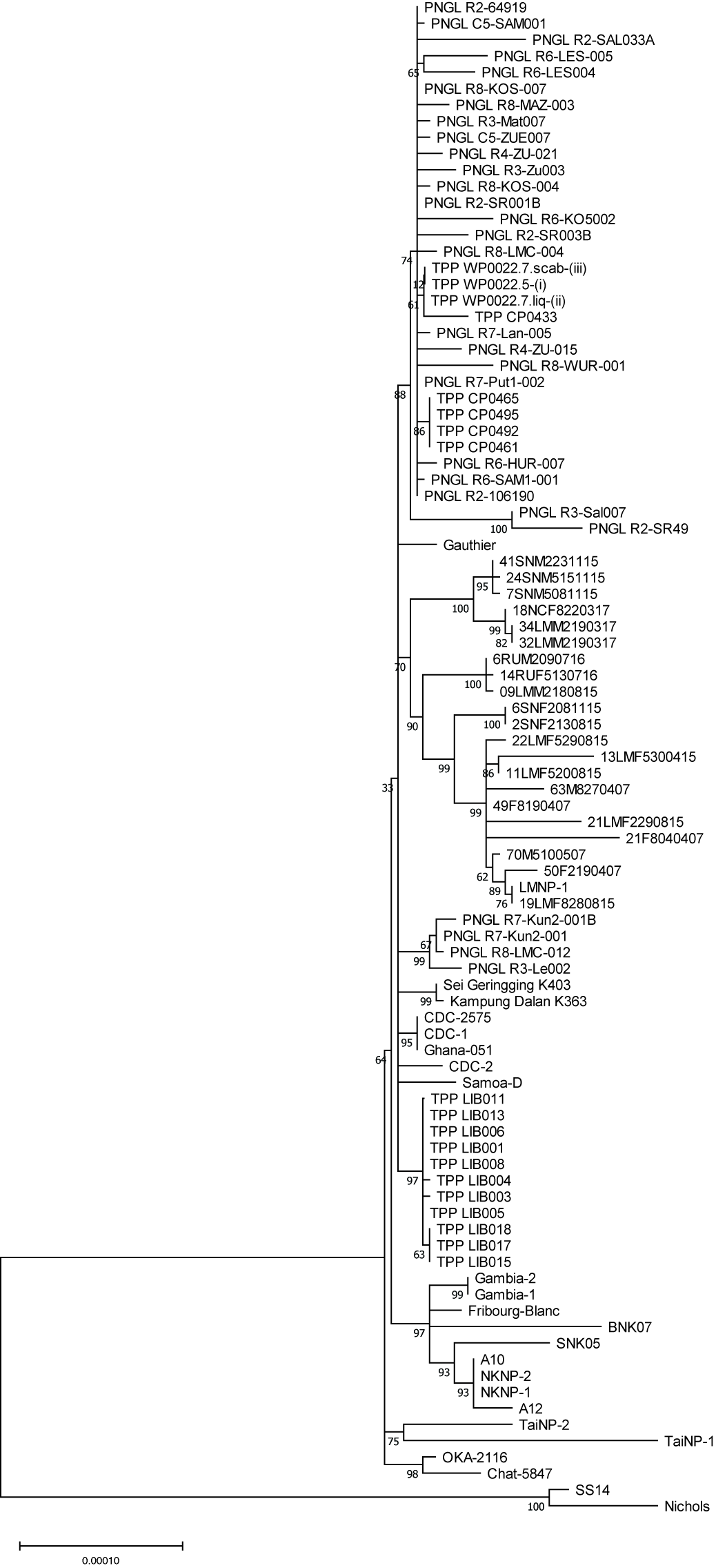

Supplement: S1 Fig — Draft genomes available from other studies were used [2,6,7,33–35]. Sequences with less than 57.8% genome coverage were not used for phylogenetic tree reconstructions. All positions containing gaps and missing data were deleted. The evolutionary history was inferred using the Maximum Likelihood method and HKY+G model [26]. As an outgroup, human syphilis genomes of strains Nichols and SS14 [32] were used. There were 93 nucleotide sequences and a total of 256,792 positions in the final dataset. Bootstrap support (1000 replicates) is shown next to the branches. The scale corresponds to the number of substitutions per nucleotide. TPE genomes from Tanzanian NHP isolates clustered together. (TIF) [file pntd.0012887.s006.tif]

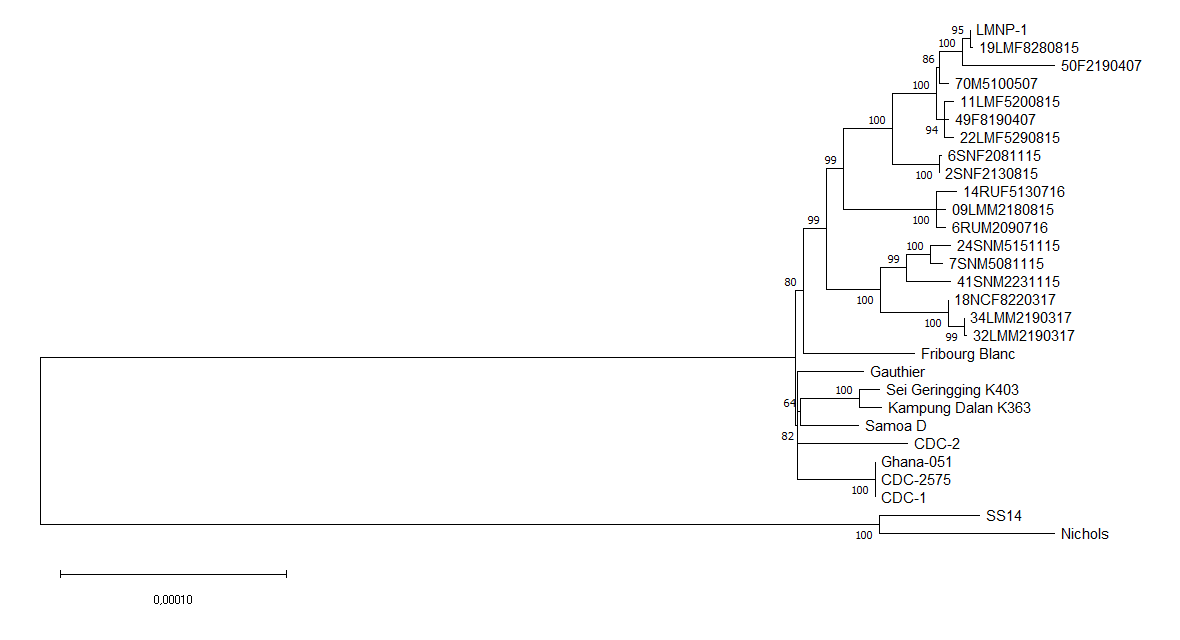

Supplement: S2 Fig — NHP TPE genomes from Tanzanian isolates. Completely sequenced genomes from African, Indonesian, and Polynesian TPE strains isolated from humans are also shown [28–31]. As an outgroup, human syphilis genomes of strains Nichols and SS14 [32] were used. The evolutionary history was inferred using the Maximum likelihood and HKY+G model [26]. All positions containing gaps and missing data were eliminated (complete deletion option). There were 873,701 positions in the final dataset. Bootstrap support (1000 replicates) is shown next to the branches. The scale corresponds to the number of substitutions per nucleotide. (TIF) [file pntd.0012887.s007.tif]

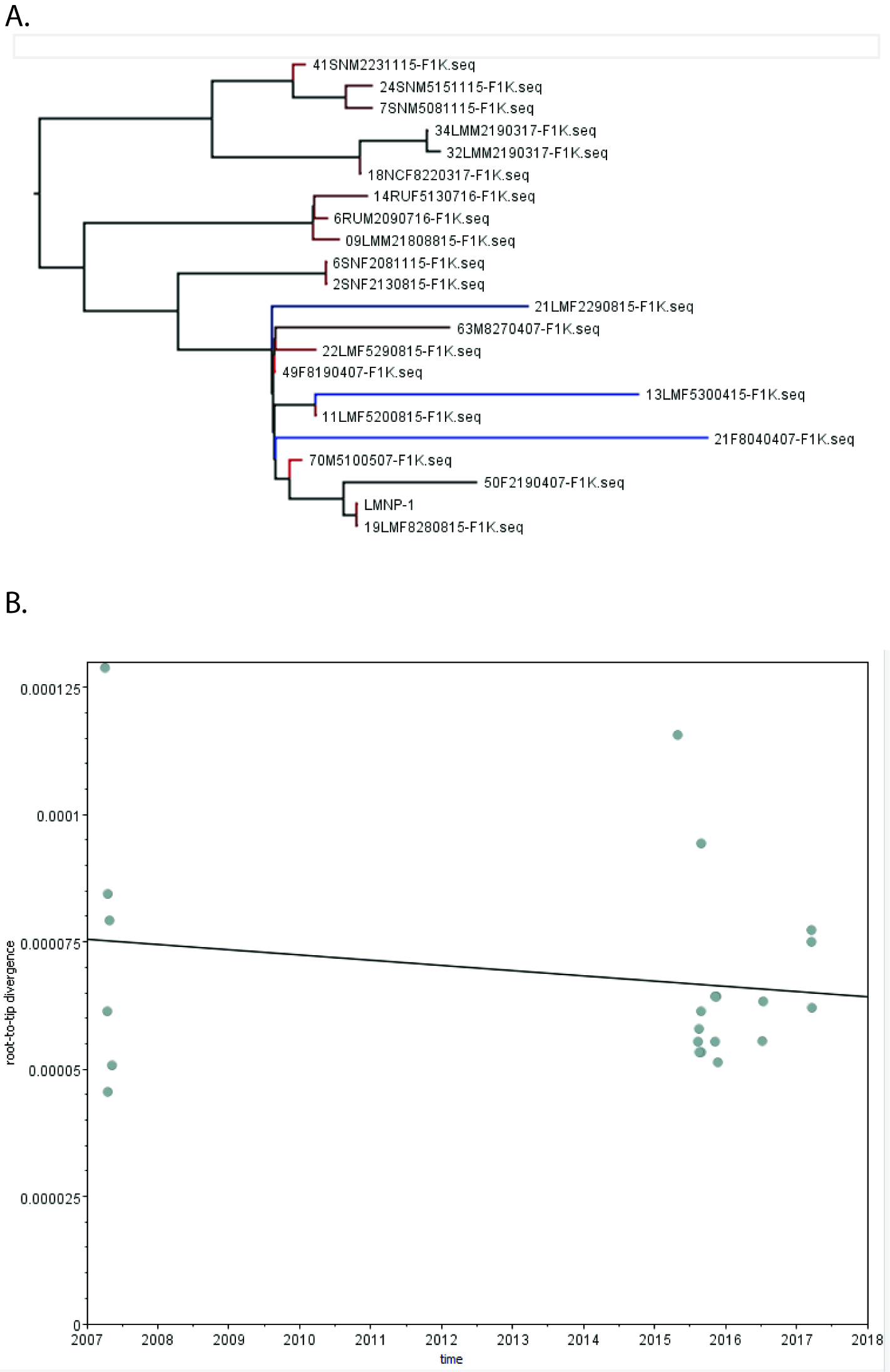

Supplement: S3 Fig — A. Phylogenetic tree. B. Root-to-tip regression graph. (TIF) [file pntd.0012887.s008.tif]

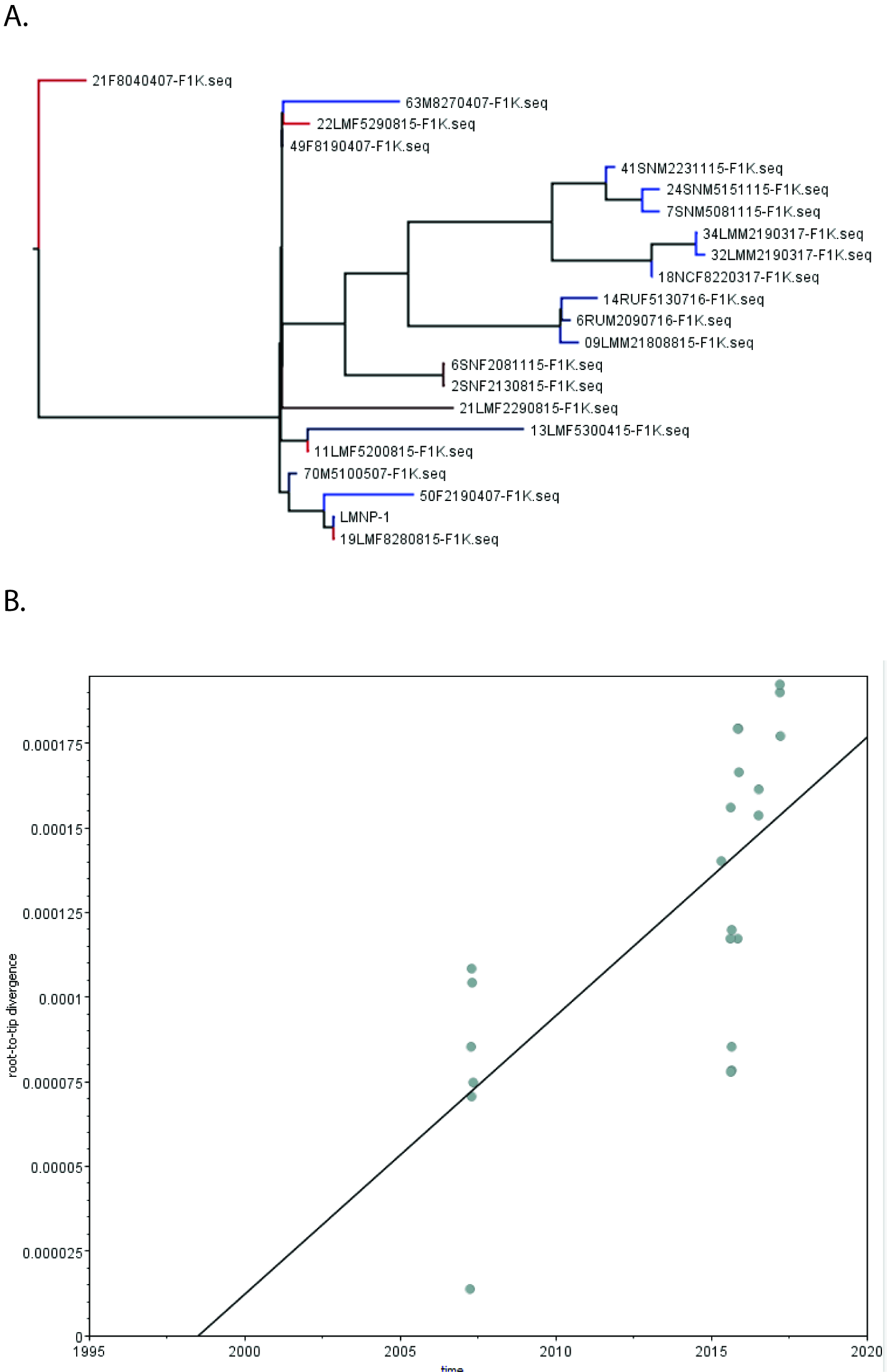

Supplement: S4 Fig — Phylogenetic tree. B. Root-to-tip regression graph. (TIF) [file pntd.0012887.s009.tif]

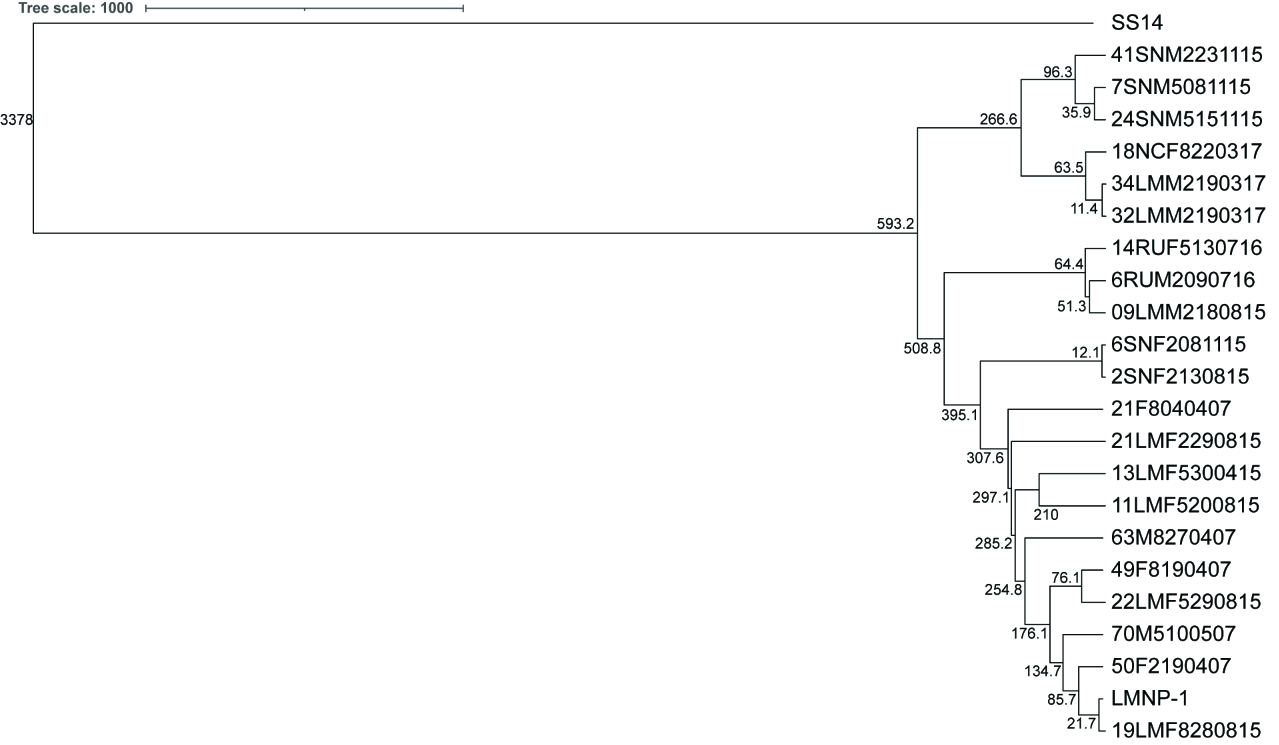

Supplement: S5 Fig — The human syphilis SS14 genome [32] was used as an outgroup. The estimated time elapsed from the most common ancestor is shown next to the branches. The length of the scale bar corresponds to 1000 years. (TIF) [file pntd.0012887.s010.tif]

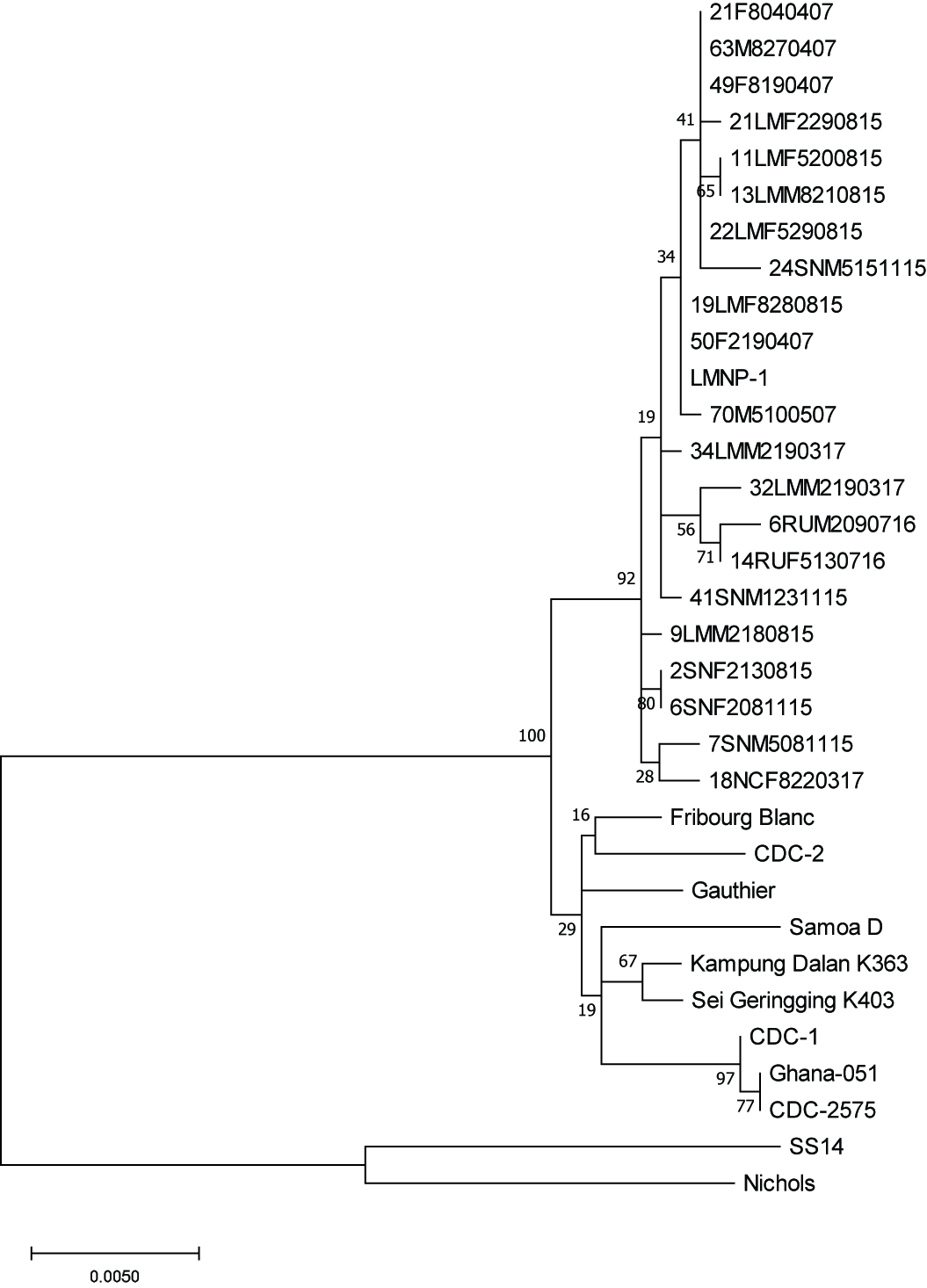

Supplement: S6 Fig — All positions containing gaps and missing data were deleted. The evolutionary history was inferred using the Maximum Likelihood method and TN93+G+I model [27]. As an outgroup, the human syphilis strains Nichols and SS14 genome [32] were used. There were 33 nucleotide sequences and a total of 1,714 positions in the final dataset. Bootstrap support (1000 replicates) is shown next to the branches. The scale corresponds to the number of substitutions per nucleotide. (TIF) [file pntd.0012887.s011.tif]
